# Misinformation of COVID-19 on the Internet: Infodemiology Study

**DOI:** 10.2196/18444

**Published:** 2020-04-09

**Authors:** Jose Yunam Cuan-Baltazar, Maria José Muñoz-Perez, Carolina Robledo-Vega, Maria Fernanda Pérez-Zepeda, Elena Soto-Vega

**Affiliations:** 1 Medicine School Universidad Anáhuac Puebla San Andres Cholula Mexico; 2 Facultad de Medicina Benemérita Universidad Autónoma de Puebla Puebla Mexico

**Keywords:** HONcode, JAMA benchmarks, DISCERN instrument, Wuhan coronavirus, COVID-19, nCoV, epidemiology, health information seeking, information quality, misinformation, public health

## Abstract

**Background:**

The internet has become an important source of health information for users worldwide. The novel coronavirus caused a pandemic search for information with broad dissemination of false or misleading health information.

**Objective:**

The aim of this study was to evaluate the quality and readability of online information about the coronavirus disease (COVID-19), which was a trending topic on the internet, using validated instruments and relating the quality of information to its readability.

**Methods:**

The search was based on the term “Wuhan Coronavirus” on the Google website (February 6, 2020). At the search time, the terms “COVID-19” or “SARS-CoV-2” (severe acute respiratory syndrome coronavirus 2) did not exist. Critical analysis was performed on the first 110 hits using the Health on the Net Foundation Code of Conduct (HONcode), the Journal of the American Medical Association (JAMA) benchmark, the DISCERN instrument, and Google ranking.

**Results:**

The first 110 websites were critically analyzed, and only 1.8% (n=2) of the websites had the HONcode seal. The JAMA benchmark showed that 39.1% (n=43) of the websites did not have any of the categories required by this tool, and only 10.0% (11/110) of the websites had the four quality criteria required by JAMA. The DISCERN score showed that 70.0% (n=77) of the websites were evaluated as having a low score and none were rated as having a high score.

**Conclusions:**

Nonhealth personnel and the scientific community need to be aware about the quality of the information they read and produce, respectively. The Wuhan coronavirus health crisis misinformation was produced by the media, and the misinformation was obtained by users from the internet. The use of the internet has a risk to public health, and, in cases like this, the governments should be developing strategies to regulate health information on the internet without censuring the population. By February 6, 2020, no quality information was available on the internet about COVID-19.

## Introduction

The coronavirus disease (COVID-19) is spreading globally from its epicenter in Hubei, China. The incidence and mortality rate have been difficult to calculate because milder cases are not being diagnosed; despite this, the World Health Organization (WHO) on March 5, 2020, declared that the latest global death rate for the disease was 3.4%, and about 80% of COVID-19 cases are mild. The cases are changing daily and can be tracked worldwide in almost real time by different websites like the one supported by Johns Hopkins University [1].

This new disease is caused by a virus from the Coronaviridae family, identified in people exposed to seafood and wild animals in a local market. Researchers in the university in Guangzhou, China, have suggested that pangolins, a mammal used in traditional Chinese medicine, could be the intermediate vector between bats and humans, because the severe acute respiratory syndrome coronavirus 2 (SARS-CoV-2) genome sequence is 99% similar to the bat coronavirus according to Zhang et al [2].

In the Munich security conference that occurred on February 15, 2020, the general director of WHO commented, “We´re not just fighting and epidemic; we’re fighting an infodemic.” It is clear that there is no way to prevent the spread of COVID-19, but it is important to verify the information on the internet to prevent the panic and misinformation associated with the disease. The fake news spreads faster than the virus. The internet is the main information source worldwide; currently 2 billion people have access to it. Online health information has grown since the 1990s, becoming popular among nonhealth personnel users; nevertheless, most of the information on the internet is unregulated, and its quality remains questionable. For users with nonmedical education, it is difficult to judge the reliability of health information on the internet. Therefore, the need for critical evaluation has taken a new dimension, and indicators of importance and quality of the content have been developed.

The likelihood that a person will view a particular website is influenced by its order of appearance on major search engines, and, in some cases, this can also be influenced if they are paid sites. It has been shown by many authors that most of the users do not go beyond the first 2 pages of citations (20-40 links) that they find [3]. The most popular search engine worldwide is Google, and it ranks its search results based on link popularity, which means that for any website, the number of hyperlinks pointing to it from other web pages will improve its rank in Google search [4].

Due to the importance of internet health searches nowadays for health personnel and nonhealth personnel, scoring systems or quality evaluation tools have been developed as a set of indicators applied to a website to provide a quality score. The most used scoring systems nowadays are the Health on the Net Foundation Code of Conduct (HONcode), the Journal of the American Medical Association (JAMA) benchmarks, and the DISCERN instrument [5-7]. Eysenbach et al [8], reported that 70% of websites presenting care information had significant quality issues. The greatest problem of the internet health information is finding valid and reliable information [8].

The HONcode is a nonprofit and nongovernmental organization that promotes transparent and reliable health information online. It is a certification of the websites based on an “ethical standard aimed at offering quality health information”. The HONcode was founded under the auspices of the Geneva Department of Employment, Social Affairs and Health in 1995. It is a code used and approved by the Economic and Social Council and the WHO. It is also one of the first URLs used as a guide to reliable sources of health care information on the internet. The HONcode consists of a minimum mechanism to provide quality, objective, and transparent medical information to the internet users. The website may display the HONcode seal if they agree to comply with the standards listed, and they are subjected to random audits for compliance [9].

The JAMA benchmarks were published in 1997. According to Silberg et al [10], it is a set of four criteria designed to assess and evaluate the quality of health information on the internet. These benchmarks are authorship, attribution, disclosure, and currency. This tool lets the reader easily decide if the site has the basic components like transparency and reliability [11].

The DISCERN instrument is a valid and reliable tool to evaluate health information. It is the first standardized quality index and was created by the Division of Public Health and Primary Health Care at Oxford University, London. It is a valid and reliable 16-point questionnaire to aid health consumers and information providers in evaluating the quality of health information on any website [12].

The Google rank, or page rank, is an algorithm developed in 2002 used by Google to give a numeric value to websites depending on the number of times that other websites are directed to a particular site, and this determines a webpage’s importance. This was one of the first tools used by Google to define the importance of websites, and currently, the algorithms are public [11].

**Figure 1:**
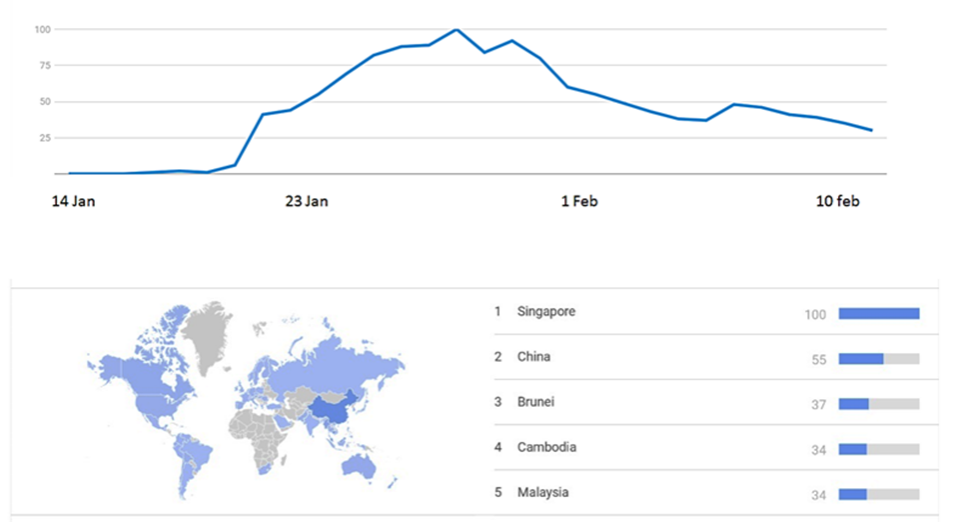
Data obtained through the Google Trends tool with the search term “Wuhan Coronavirus” between January 14 and February 14. The map shows the world trend of the searched terms on the same dates by country. Figures were obtained from Google Trends.

Currently, COVID-19 has been a trending topic worldwide. Around January 10, 2020, most of the news around the world talked about a new coronavirus strain that started in China and was spreading fast. This created an avalanche of search for information on the internet called an “infodemic.” In a few days, the network was filled with information, sometimes with accurate content and sometimes with fake content that pointed toward the possibility of becoming infected even after receiving regular mail from China [13]. By the end of January 2020 (20 days later), this infodemic increased, as the new disease had become a trending topic with the maximum search for a term reported by Google according to Google Trends, especially after the WHO declared COVID-19 as a global health emergency on January 31, 2020 (Figure 1) [14].

In this work, we evaluate the quality of online health information that internet users found about COVID-19 at the beginning of the epidemic from January until February 6. The search was performed using “Wuhan” and “Coronavirus” as keywords because, at that moment, these were the most popular keywords, and the objective was to evaluate what nonhealth personnel users found on the network. By February 6, 2020, the terms COVID-19 or SARS-CoV-2 were still not established.

## Methods

### Search Strategy

The search terms “Coronavirus” and “Wuhan” were used (February 6, 2020) on the Google search engine (google.com). The search was done using an updated browser of Google Chrome version 79.0.3945.130. We accessed Google from the University Anáhuac Puebla at Tlaxcalancingo, Puebla, México

Before the search, all existing cookies were deleted from the browser, and the Google settings were used to establish the English language as a condition.

We performed one search and the first 110 websites obtained were shared with the observers, who worked with each website. Websites that were not in the English or Spanish language were excluded. All the instruments were assessed by four independent observers for each website, and any disagreements were resolved by consensus prior to the final analysis.

The Google search engine itself was evaluated as part of the critical assessment and not just the landing page of the Google search results. Therefore, if further information was obtained elsewhere on the website via subheadings, links, or leading pages, this information was obtained as a result of being directed to it, either directly or indirectly, via the *original* Google search.

### Quality Assessment Instruments

Quality evaluation tools have been developed to assess health information using various criteria. Amongst the tools available, we selected three different validated evaluation tools, the HONcode, the JAMA benchmarks, and the DISCERN tool.

### HONcode

The HONcode is based on an 8-point code of conduct comprising of authority, complementarily, confidentiality, attribution, justifiability, transparency of authorship, financial disclosure, and advertising policy. Any website that complies with this code is granted permission to display the HON award-like badge on its website. The certificate is valid only for 1 year. The HONcode is the oldest quality evaluation tool being used to date [9]. To evaluate the HONcode, we downloaded its software, and, for each link, we searched for the seal.

### JAMA Benchmarks

The JAMA benchmarks evaluate the following points: authorship (authors and contributors, their affiliations, and relevant credentials should be displayed), the attribution (clear references and sources for all content should be provided), the disclosure (ownership of the website, the sponsorship, the advertising, the underwriting, the commercial funding or support sources, and any potential conflicts of interest), and currency (dates of initial posting and updating of the content should be noted) [10]. For each criteria (authorship, attribution, currency, and disclosure) the website received 1 point; the range was from 0 to 4 points.

### DISCERN Instrument

The DISCERN instrument comprised 3 sections, the first 2 assesses the reliability and the quality of the written information. The third section rates the publication as a whole. Each question is scored on a range from 1 (definite no) to 5 (definite yes). A score of 2 and 4 is a range given for cases in which the criterion is partially met to some extent. The maximum total score is 80, and the quality of each website is classified as high (≥65 points), moderate (33-64 points), or low (16-32 points) [12]. To evaluate the DISCERN score, we designed a Microsoft Excel page where a row was assigned to each question of the instrument. Each website was evaluated, and the value of each question was introduced manually into the corresponding cell; the score for each question was from 1 to 5. For the 16th question, the function of mode was used with rows 1-15. The 17th row was the addition of rows 1-16, and that was the DISCERN value of the website.

### Google Rank

Google Rank, or page rank, uses the URL of the site and the keyword used. The algorithm then determines the position number of the website. In this study, two free use rank sites were used [15,16]. They were used by entering the URL of each of the 110 sites and the same keywords that were used in the search: “coronavirus” and “Wuhan.”

### Categorization

The websites reviewed were categorized based on affiliation (commercial, news, university or medical center, a nonprofit organization, or government), content type (medical facts, clinical trials, human interest stories, and questions and answers), and specialization of topic and content (website exclusively related to coronavirus or only part of the website).

### Contrast to Medical Bibliography

From the results, the main ideas of the first 50 websites were compared to the medical literature available on PubMed, considering main ideas as all the facts mentioned on a website (eg, days between contagion and the onset of symptoms, genomic characteristics of the virus, recommendations to prevent contagion among others). The information was classified as true (if everything on the website was found in any published paper found in PubMed), partially true (if most of the information on the site was found in one or more papers published and found in PubMed, but there is still missing information), or false (if everything on the site was not found in any published article in PubMed). We avoided information on the number of cases and territorial virus expansion because this information could quickly change. The websites in which there was no health information to discuss, non-free-access websites, and websites considered medical literature were excluded.

### Statistics

Quantitative analysis of the database was done. Besides comparisons of the values obtained in JAMA and DISCERN scores between the first 50 websites, the rest of the comparisons were determined using an unpaired *t* test. The statistical analysis was performed using the GraphPad Prism software.

## Results

### Google Trends

As previously mentioned, according to Google Trends, the search for coronavirus in the last 30 days was observed as is shown in Figure 1. It reached its maximum value on January 30; during this period of 30 days, the search was also a trending topic. The Google Trends also showed the behavior on a map, where countries with the highest levels of the search were highlighted. The more searched keywords according to Google Trends were “Coronavirus,” “outbreak epidemic,” “gross death rate,” “Coronavirus symptoms,” and “Coronavirus and China.”

The Google search for COVID-19 retrieved 309,000,000 results, and the first 110 websites were critically analyzed (Multimedia Appendix 1).

### HONcode

The analysis of the HONcode showed that from the survey of 110 websites, only 1.8% (2 websites) had the HONcode seal (Table 1).

**Table 1:** Results of the analysis of the 110 websites consulted.

Variables		Websites, n
**HONcode^a^**		
	Certified	2
	Not certified	108
**JAMA^b^**		
	0	43
	1	26
	2	19
	3	11
	4	11
**DISCERN score**		
	High (≥65)	0
	Moderate (33-64)	33
	Low (16-32)	77
**Categorization or affiliation**		
	News	61
	Commercial	21
	Nonprofit organization	5
	Government	9
	University	0
	Medical center	1
	Nonprofit organization or government	3
	University or medical center	8
	News or commercial	1
	University or medical center and nonprofit organization	1
**Exclusivity**		
	Partly exclusive	61
	Exclusive	49
**Subtype or content**		
	Medical facts	11
	Question and answer	10
	Human interest stories	43
	Clinical trials	0
	Medical facts and question and answers	5
	Medical facts, human-interest stories, and question and answer	5
	Medical facts and human-interest stories	27
	Medical facts and clinical trial	3
	Human-interest stories and question and answer	6
**Language**		
	English	103
	Spanish	7

^a^HONcode: Health on the Net Foundation Code of Conduct.

^b^JAMA: Journal of the American Medical Association.

### JAMA Benchmarks

The JAMA benchmark analysis showed that, of the 110 websites, 39.1% (43 websites) did not fit any of the JAMA benchmark criteria, 23.6% (26 websites) achieved only 1 criterion, 17.3% (19 websites) achieved 2 criteria, 10.0% (11 websites) achieved 3 criteria, and 10.0% (11 websites) achieved all 4 criteria (Table 1).

On average, all the websites achieved a mean of 1.28 (SD 1.34) criteria; the first half of websites achieved a mean of 1.95 (SD 1.35) and the second half achieved a mean of 0.68 (SD 0.95) criteria. There was a significant difference between the first half and the second half (*P*<.001).

Of the 43 websites that did not achieve any of the JAMA benchmark criteria, 9 appeared in the first 50 websites. In addition, from the 11 websites that achieved four criteria, 10 were found on the first 50 websites, suggesting that the quality of the information may reduce after the first 55 websites.

### DISCERN Score

The DISCERN score for the analyzed websites’ results are as follows. Of the 110 websites, a high score (65 or more points) was not achieved by any of the websites, a moderate score (33-64) was achieved by 30.0% (n=33) of the websites, and a low score (16-32 points) was achieved by 70.0% (n=77) of the websites (Table 1).

On average, all websites achieved a mean score of 28.91 (SD 10.34). The first half of the websites achieved a mean score of 24.36 (SD 8.36), and the second half achieved a mean score of 33.43 (SD 10.21). There was a significant difference between the first half and the second half (*P*<.001).

### Google Rank

The Google ranking yielded 7 websites with a ranking position for “Coronavirus” and 5 websites with a ranking position for “Wuhan”; only 2 websites had rankings for both keywords (website 1 and 28 in Multimedia Appendix 1). The best ranked website for the word “Wuhan” was the first website (Multimedia Appendix 1), and for the keyword “Coronavirus” it was the second website, which was also ranked in the top 10 websites of the Google ranking. Only 9.1% (n=10) of the 110 visited websites had a position in the Google ranking for one or both keywords in the top 100 positions.

### Website Categorization

The analysis on the website categorization or affiliation showed that, of the 110 websites visited, 56.4% (n=62) were on general news pages, 19.1% (n=21) were on commercial pages, 8.2% (n=9) were on pages associated with a government, 7.3% (n=8) were on pages considered nonprofit organizations, and only 0.9% (n=1) were on the pages associated with universities or medical websites.

Of the 110 websites reviewed, 44.5% (n=49) of them presented exclusive information about the coronavirus, while 55.5% (n=61) presented it as part of the notes on the website.

Despite the fact that most of the sites were not specialized in medicine, 39.1% (n=43) of the information presented was considered health information; the rest of the websites presented epidemiological data, stories about the patients, or how people were living through the epidemic.

Language analysis showed that 92.7% (n=102) of the pages had English as their main language (Table 1).

### Comparison to Medical Bibliography

The main ideas found in the text of the first 50 websites were analyzed to compare with the information from the medical bibliography. Website numbers 3, 10, 13, 22, 32, 33, 34, 39, 42, 43, 46, and 48 in Multimedia Appendix 1 were excluded, as there were no main ideas to compare with the medical bibliography. Website number 18 was excluded since it was not free access. Website 26 was excluded because it was considered medical literature.

From the remaining 36 websites, 15 had main ideas considered “True,” 16 had main ideas considered “Partially true,” and 5 had main ideas considered “False” compared to the medical literature present in PubMed at that specific time [17-24] (Multimedia Appendix 2).

## Discussion

Due to the novelty of the disease, it was a trending topic by February 6, 2020. Google Trends reported it with a 100 factor before the Coronavirus had its final name, COVID-19 or SARS-CoV-2. It was not until Tuesday, February 20, that the WHO agency announced the official name as COVID-19. This name was chosen to avoid indicating a geographical location, animal species, or human ethnic group [25].

Most of the information that the internet users got came from news sources, representing 56.4% (62/110) of the websites returned by Google. At best, this news presented a summary interpretation of the statements from the health personnel involved in the treatment of the patients or information provided from health organizations like WHO. The infodemic at this time was that there was no information with clear scientific basis.

The evaluation of the quality of health information presented by the first 110 websites retrieved by the Google search engine showed that only 2 websites have the HONcode, 11 websites achieved the four JAMA benchmark criteria, and none of the websites were evaluated as excellent with the DISCERN instrument.

According to the Google ranking, the most influential websites were in English and appeared in the first 3 links displayed; although there was no direct relation between the position in the Google ranking and the site content’s quality. The Google ranking might be influenced by the country where the search was performed (Mexico); COVID-19 was not present and people with no medical training were looking at news sites. From the website analysis of the health information quality at the time of the search, it became clear that the information provided by the Google search engine did not have the quality standards required for health information, and it was not entirely reliable. The excess of poor-quality information without scientific support from the news and social media increased the interest in the information search, putting the world on alert for a possible pandemic that would cause many deaths, alerting users about an unknown virus, and presenting cataclysmic images.

It is important to emphasize that the internet users are responsible for the quality of information they obtain from websites. Nowadays, misinformation is an important problem; people do not tend to critically assess the information they read and often when making important decisions regarding their lives and health. The misinformation is associated with panic shopping, buying medical supplies or drugs, and, even worst, taking drugs without a medical prescription. The misinformation impact can be devastating, social media providers are trying to filter the fake news, but this has not stopped the conspiracy theorists, swindlers, and liars on the internet. The financial markets and governments are looking to avoid panic. The scientific information about COVID-19 flows freely in the networks like never before, but it must be accompanied with a proper interpretation by the media and internet users. In countries where drugs are sold without a prescription, people read clinical trials on social media and go to the pharmacy to buy all the drugs in stock as if it were toilet paper.

The internet is the most powerful force disrupting the news; the internet shifts the power from governments to society, and it is society who is pressuring the governments to make decisions, sometimes based on fake news. During the COVID-19 pandemic, it has been difficult for governments and search engines to control the quality and flow of information concerning the experiences of this pandemic. It is clear that governments as well as institutions like WHO must work together to create guidelines and control mechanisms over the information flow on the internet and establish global ethical codes under which health information can be published, as it also affects the politics and economies of the countries. It is also important to consider that some part of the population may prefer to receive information by other methods than the internet, such as radio, television, or newspapers. In 2019, It was estimated that only 53.9% of the world population has internet access, leaving the rest, mainly in the third world, without the tool of information searching [3].

To prevent inadequate responses and fears from the population, it is important that governments develop a strategy to teach their residents how to verify the quality of what they read, especially in the case of health information. Every day, the number of users looking for their diagnosis and treatment on the internet increases, making the internet a two-edged tool for the health sector. Government agencies should consider the use of a regulatory mechanism to control false or misleading health information. False health information can cause significant social harm by feeding false concepts of disease. In addition, health personnel must assume a role in society with these 5 recommended actions: (1) don’t share information if its veracity has not been proven; (2) participate on mass media programs to share legitimate information; (3) promote hygiene actions and vaccination; (4) educate patients to identify alarm symptoms and instruct them on what to do if these symptoms appear; (5) produce media content and promote websites of academic institutions.

The governments and health organizations like the WHO should take an active role of information on cases like the COVID-19 pandemic. Some of the actions that should be considered to spread correct and reliable information on the internet amongst their populations are to share reliable information or suggest some sources of reliable information on the government’s websites, subsidize more visibility of reliable information on massive search engines, subsidize scientific institutes or organizations to share reliable information, develop a tool where health personnel may assess the quality of information on websites, and use these assessments to find reliable information.

## Appendix

Multimedia Appendix 1 Supplementary file 1.

Multimedia Appendix 2 Supplementary file 2.

## Abbreviations

COVID-19: coronavirus disease
HONcode: Health on the Net Foundation Code of Conduct
JAMA: Journal of the American Medical Association
SARS-CoV-2: severe acute respiratory syndrome coronavirus 2
WHO: World Health Organization
