# Supplementary material for: Misinformation of COVID-19 on the Internet: Infodemiology Study
Source: JMIR Public Health Surveill. 2020 Apr 9;6(2):e18444. doi: 10.2196/18444 (PMC7147328; doi:10.2196/18444)
Supplement: Multimedia Appendix 1 [file publichealth_v6i2e18444_app1.docx]

Table 1. Quality health information analysis of the first 110 websites, using the Google search engine with the key words “Wuhan Coronavirus”.

| **No.** | **HON code** | **JAMA benchmark** | **DISCERN score** | **Categorization/ affiliation** | **Website** | **Sub-type/content** | **Language** | **Google Rank** | **Website link** |
| --- | --- | --- | --- | --- | --- | --- | --- | --- | --- |
| 1 | No | 4 | 23 | News | Partly exclusive | Medical facts, question and answer | English | 7/1 | https://www.nytimes.com/article/what-is-coronavirus.html |
| 2 | No | 2 | 19 | News | Partly exclusive | Medical facts, question and answer | English | 10/0 | https://www.aljazeera.com/news/2020/01/china-coronavirus-death-toll-surges-latest-updates-200131232932230.html |
| 3 | No | 2 | 19 | News | Exclusive | Human interest stories | English | 10/0 | https://www.aljazeera.com/news/2020/02/wuhan-turns-social-media-vent-anger-coronavirus-response-200203085324619.html |
| 4 | No | 3 | 61 | Commercial | Exclusive | Medical facts | Spanish | 0/0 | https://es.wikipedia.org/wiki/coronavirus_de_wuhan |
| 5 | No | 2 | 38 | Non-profit organization | Exclusive | Medical facts, | English | 0/0 | https://en.wikipedia.org/wiki/2019%e2%80%9320_wuhan_coronavirus_outbreak |
| 6 | No | 2 | 38 | News | Partly exclusive | Medical facts, human interest stories, question and answer | English | 0/0 | https://www.bbc.com/news/health-51345279 |
| 7 | No | 1 | 43 | Non-profit organization, government | Exclusive | Medical facts, question and answer | English | 2/0 | <https://www.who.int/emergencies/diseases/novel-coronavirus-2019> |
| 8 | No | 3 | 27 | News | Partly exclusive | Medical Facts | English | 0/0 | <https://www.businessinsider.com/wuhan-coronavirus-research-studies-published-2020-1> |
| 9 | No | 3 | 19 | News | Partly exclusive | Medical facts, human interest stories | English | 0/0 | <https://www.theguardian.com/world/live/2020/jan/29/coronavirus-live-updates-china-wuhan-death-toll-cases-symptoms-treatment-evacuation-us-japanese-citizens-latest-news> |
| 10 | No | 3 | 19 | News | Partly exclusive | Human interest stories | Spanish | 0/0 | <https://www.eluniversal.com.mx/mundo/coronavirus-british-airways-y-american-airlines-suspenden-vuelos-china> |
| 11 | No | 4 | 29 | News | Exclusive | Human interest stories, question and answer | English | 9/0 | https://www.cnet.com/how-to/coronavirus-cases-pass-11000-us-declares-emergency-everything-we-know/ |
| 12 | Yes | 2 | 25 | Non-profit organization | Exclusive | Question and answer | English | 0/0 | https://familydoctor.org/condition/coronavirus/ |
| 13 | No | 4 | 17 | News | Exclusive | Human interest stories | English | 0/0 | https://foreignpolicy.com/2020/02/05/coronavirus-epidemic-wuhan-misinformation-online-social-media/ |
| 14 | No | 2 | 37 | Non-profit organization, government | Partly exclusive | Medical facts, human interest stories | English | 0/4 | <https://www.cdc.gov/coronavirus/2019-ncov/index.html> |
| 15 | No | 1 | 30 | Non-profit organization, government | Partly exclusive | Question and answer | English | 21/0 | <https://www.ecdc.europa.eu/en/novel-coronavirus-china> |
| 16 | No | 4 | 23 | News | Exclusive | Question and answer | English | 0/0 | <http://theconversation.com/how-contagious-is-the-wuhan-coronavirus-and-can-you-spread-it-before-symptoms-start-130686> |
| 17 | No | 4 | 22 | News | Exclusive | Medical facts, question and answer, human interest stories | English | 0/17 | https://time.com/5759289/wuhan-pneumonia-outbreak-disease/ |
| 18 | No | 3 | 23 | News/Commercial | Exclusive | Human interest stories | English | 0/0 | <https://www.wsj.com/articles/united-american-airlines-suspend-hong-kong-service-as-coronavirus-saps-demand-11580897463> |
| 19 | No | 4 | 19 | News | Exclusive | Human interest stories, question and answer | English | 0/0 | https://geoawesomeness.com/track-china-coronavirus-real-time-map-global-cases-death-wuhan/ |
| 20 | No | 2 | 26 | News | Partly exclusive | Human interest stories, question and answer | English | 0/0 | <https://www.bbc.co.uk/news/uk-51292590> |
| 21 | No | 1 | 17 | News | Partly exclusive | Human interest stories | English | 0/0 | <https://elpais.com/elpais/2020/01/28/inenglish/1580220348_402354.html> |
| 22 | No | 3 | 17 | News | Partly exclusive | Human interest stories | English | 0/0 | <https://www.france24.com/en/20200128-chinese-tourist-in-serious-condition-in-france-s-fourth-coronavirus-case> |
| 23 | No | 1 | 17 | Government | Exclusive | Human interest stories | English | 0/0 | <http://www.xinhuanet.com/english/2020-01/29/c_138741063.htm> |
| 24 | No | 2 | 23 | News | Exclusive | Question and answer | English | 0/0 | <https://www.dw.com/en/coronavirus-everything-you-need-to-know/a-52102486> |
| 25 | No | 1 | 25 | Government | Exclusive | Question and answer | English | 0/0 | <https://www.rivm.nl/en/novel-coronavirus-in-china> |
| 26 | No | 4 | 45 | Medical Center | Partly exclusive | Medical facts, clinical trials | English | 0/0 | <https://www.thelancet.com/journals/lancet/article/piis0140-6736(20)30183-5/fulltext> |
| 27 | No | 2 | 18 | News | Partly exclusive | Human interest stories | English | 0/0 | https://www.telegraph.co.uk/news/2020/01/25/british-citizens-wuhan-left-dark-foreign-office-coronavirus/ |
| 28 | No | 0 | 32 | Government | Partly exclusive | Question and answer, medical facts | English | 78/47 | <https://www.ontario.ca/page/2019-novel-coronavirus-2019-ncov> |
| 29 | No | 4 | 23 | News | Exclusive | Question and answer | English | 0/0 | https://www.forbes.com/sites/leahrosenbaum/2020/01/23/everything-you-need-to-know-about-the-wuhan-coronavirus-outbreak/ |
| 30 | No | 4 | 20 | News | Partly exclusive | Human interest stories, question and answer | English | 0/0 | <https://www.cnn.com/2020/01/22/asia/china-wuhan-coronavirus-deadly-intl-hnk/index.html> |
| 31 | No | 0 | 24 | Government | Exclusive | Question and answer | English | 0/0 | <https://multco.us/health-officer/wuhan-coronavirus> |
| 32 | No | 4 | 21 | News | Partly exclusive | Human interest stories | English | 0/0 | <https://www.dailymail.co.uk/health/article-7942251/britons-coronavirus-airlift-wuhan-quarantined.html> |
| 33 | No | 1 | 23 | News | Exclusive | Human interest stories, question and answer | English | 0/0 | <https://www.euronews.com/2020/01/22/watch-live-who-director-general-statement-on-coronavirus-outbreak> |
| 34 | No | 1 | 28 | Government | Exclusive | Question and answer | English | 0/0 | <https://thl.fi/web/infectious-diseases/what-s-new/wuhan-coronavirus-latest-updates> |
| 35 | No | 2 | 30 | University/medical center | Partly exclusive | Question and answer | English | 0/0 | <https://www.ed.ac.uk/health-safety/guidance/communicable-infectious-diseases/wuhan-coronavirus> |
| 36 | No | 3 | 29 | Government | Partly exclusive | Question and answer | English | 0/0 | https://www.weforum.org/agenda/2020/01/wuhan-coronavirus-china-cepi-vaccine-davos/ |
| 37 | No | 0 | 21 | Non-profit organization | Exclusive | Human interest stories | English | 0/0 | https://globalvoices.org/specialcoverage/how-will-the-wuhan-coronavirus-impact-the-political-future-of-china/ |
| 38 | No | 1 | 20 | News | Exclusive | Human interest stories | English | 0/0 | https://phw.nhs.wales/news/public-health-wales-response-to-outbreak-in-wuhan-china/ |
| 39 | No | 2 | 22 | Commercial | Exclusive | Medical facts | English | 0/0 | https://blogs.sas.com/content/graphicallyspeaking/2020/02/03/improving-the-wuhan-coronavirus-dashboard/ |
| 40 | No | 1 | 18 | News | Partly exclusive | Human interest stories | English | 0/0 | <https://www.voanews.com/science-health/new-tech-sharp-docs-made-fast-id-wuhan-coronavirus-possible> |
| 41 | No | 2 | 24 | News | Partly exclusive | Human interest stories | English | 0/0 | https://www.asiatimes.com/2020/01/opinion/how-china-failed-to-manage-wuhan-coronavirus/ |
| 42 | No | 0 | 20 | Government | Exclusive | Medical facts | English | 0/33 | <https://www.moh.gov.sg/2019-ncov-wuhan> |
| 43 | No | 0 | 16 | Non-profit organization | Partly exclusive | Human interest stories | English | 0/0 | https://aleteia.org/2020/02/01/prayer-to-protect-against-the-wuhan-coronavirus/ |
| 44 | No | 1 | 24 | Commercial | Exclusive | Question and answer | Spanish | 0/0 | https://www.practicaespanol.com/en/13-clear-things-about-wuhans-coronavirus-in-china/ |
| 45 | No | 0 | 29 | Government | Exclusive | Medical facts, clinical trials | English | 0/0 | https://www.imperial.ac.uk/mrc-global-infectious-disease-analysis/news--wuhan-coronavirus/ |
| 46 | No | 1 | 16 | News | Partly exclusive | Medical facts | English | 0/0 | <https://www.voacambodia.com/a/where-wuhan-coronavirus-is-spreading/5262286.html> |
| 47 | No | 1 | 24 | University/medical center | Partly exclusive | Medical facts | English | 0/0 | <https://www.ntu.edu.tw/english/spotlight/2020/1797_20200130.html> |
| 48 | No | 1 | 16 | News | Exclusive | Human interest stories | English | 0/0 | <https://www.helsinkitimes.fi/finland/finland-news/domestic/17271-first-case-of-wuhan-corona-virus-confirmed-in-finland.html> |
| 49 | No | 1 | 22 | News | Partly exclusive | Medical facts, human interest stories | English | 0/0 | <http://ba.n1info.com/english/news/a406863/wuhan-coronavirus-continues-its-global-spread-with-more-than-6-000-cases.html> |
| 50 | No | 3 | 21 | News | Partly exclusive | Human interest stories | English | 0/0 | <https://www.latimes.com/science/story/2020-01-28/wuhan-chinas-coronavirus-50-million-people-quarantined> |
| 51 | No | 3 | 24 | News | Partly exclusive | Human interest stories | English | 0/0 | https://www.asiatimes.com/2020/01/opinion/how-china-failed-to-manage-wuhan-coronavirus/ |
| 52 | No | 0 | 18 | News | Partly exclusive | Human interest stories | English | 0/0 | <https://www.lrt.lt/en/news-in-english/19/1137526/2-lithuanians-currently-in-wuhan-amid-coronavirus-outbreak-mfa> |
| 53 | No | 2 | 16 | Commercial | Partly exclusive | Question and answer | English | 0/0 | <https://www.malaysiaairlines.com/au/en/advisory/china-coronavirus.html> |
| 54 | No | 0 | 20 | News | Exclusive | Human interest stories | English | 0/0 | <https://www.efe.com/efe/english/world/china-sends-thousands-of-medical-staff-to-wuhan-as-coronavirus-toll-rises/50000262-4160253> |
| 55 | No | 0 | 20 | News | Exclusive | Medical facts, human interest stories | English | 0/0 | <http://hr.n1info.com/english/news/a479163/wuhan-coronavirus-continues-its-global-spread-with-more-than-6-000-cases.html> |
| 56 | No | 2 | 28 | News | Partly exclusive | Human interest stories | English | 0/0 | https://qz.com/1791611/wuhan-coronavirus-chinese-people-channel-anger-through-chernobyl/ |
| 57 | No | 3 | 35 | News | Partly exclusive | Human interest stories | English | 0/0 | <https://www.thedenverchannel.com/news/local-news/colorado-native-quarantined-in-wuhan-china-due-to-coronavirus> |
| 58 | No | 1 | 40 | Commercial | Exclusive | Human interest stories | English | 0/0 | https://factcheck.afp.com/chinese-doctors-have-not-projected-11-million-people-quarantined-wuhan-china-will-die-coronavirus |
| 59 | No | 1 | 38 | News | Partly exclusive | Medical facts, human interest stories | English | 0/0 | https://www.businessinsider.sg/china-coronavirus-wuhan-hubei-medics-infections-outstrip-sars-2020-1/ |
| 60 | No | 0 | 39 | Commercial | Partly exclusive | Medical facts, human interest stories | English | 0/0 | <https://mainichi.jp/english/articles/20200128/p2g/00m/0na/089000c> |
| 61 | No | 0 | 40 | News | Partly exclusive | Human interest stories | English | 0/0 | https://www.dutchnews.nl/news/2020/01/coronavirus-latest-dutch-look-into-bringing-expats-in-wuhan-home/ |
| 62 | No | 0 | 35 | News | Partly exclusive | Human interest stories | Spanish | 0/0 | <https://laverdadnoticias.com/mundo/este-es-el-mercado-de-wuhan-donde-se-habria-originado-el-coronavirus-fotos-20200126-0153.html> |
| 63 | No | 0 | 40 | university/medical center | Partly exclusive | Medical facts | English | 0/0 | <https://www.medscape.com/viewarticle/924268> |
| 64 | No | 0 | 44 | Commercial | Exclusive | Medical facts, human interest stories | English | 0/0 | https://www.siasat.com/coronavirus-over-75000-infected-wuhan-1813095/ |
| 65 | No | 2 | 42 | News | Exclusive | Medical facts, human interest stories | English | 0/0 | https://www.citynews1130.com/2020/01/25/public-health-officials-in-ontario-call-evening-news-conference/ |
| 66 | No | 1 | 39 | News | Partly exclusive | Human interest stories | English | 0/0 | https://thepienews.com/news/novel-coronavirus-international-students/ |
| 67 | No | 1 | 38 | University/medical center | Exclusive | Medical facts, clinical trials | English | 0/0 | http://english.shanghaipasteur.cas.cn/research2016/rp2016/202001/t20200125_229792.html |
| 68 | Yes | 1 | 51 | University/medical center | Partly exclusive | Medical facts, human interest stories | English | 0/0 | https://www.news-medical.net/news/20200128/travel-data-reveals-top-20-cities-at-risk-of-wuhan-coronavirus-spread.aspx |
| 69 | No | 0 | 40 | News | Exclusive | Medical facts, human interest stories | English | 0/0 | https://www.peruviantimes.com/27/four-people-under-observation-for-wuhan-coronavirus/32133/ |
| 70 | No | 0 | 51 | News | Exclusive | Human interest stories | English | 0/0 | https://nypost.com/2020/01/25/doctor-at-hospital-in-wuhan-dies-after-treating-patients-with-coronavirus/ |
| 71 | No | 1 | 56 | News | Partly exclusive | Medical facts, human interest stories | English | 0/0 | https://www.summitdaily.com/news/wuhan-coronavirus-is-a-concern-but-less-of-a-worry-than-the-flu-colorado-health-officials-say/ |
| 72 | No | 0 | 39 | News | Partly exclusive | Medical facts, human interest stories | English | 0/0 | https://fortune.com/2020/01/27/wuhan-coronavirus-global-markets-shutter/ |
| 73 | No | 0 | 32 | Commercial | Partly exclusive | Human interest stories, question and answer | English | 0/0 | https://give2asia.org/donate-help-fight-coronavirus-outbreak-wuhan-china/ |
| 74 | No | 0 | 40 | Commercial | Exclusive | Human interest stories | English | 0/0 | https://www.news18.com/news/world/coronacoron-outbreak-live-updates-corona-virus-symptoms-prevention-cure-china-wuhan-2473519.html |
| 75 | No | 0 | 29 | Commercial | Exclusive | Human interest stories | English | 0/0 | https://www.facebook.com/ladbible/videos/487922995254448/ |
| 76 | No | 1 | 27 | Government | Partly exclusive | Medical facts | English | 0/0 | http://mofa.gov.pk/coronavirus-in-china-update-on-welfare-of-pakistani-community-in-wuhan/ |
| 77 | No | 0 | 17 | Commercial | Exclusive | Human interest stories | English | 0/0 | https://www.flyroyalbrunei.com/hong-kong-sar/en/coronavirus-pneumonia-in-wuhan-china/ |
| 78 | No | 0 | 33 | university/medical center | Partly exclusive | Medical facts, human interest stories, question and answer | Spanish | 0/0 | https://www.actasanitaria.com/coronavirus-de-wuhan-china-2019-ncov-lo-que-tiene-que-saber-para-evitar-una-epidemia-de-panico/ |
| 79 | No | 4 | 33 | News | Partly exclusive | Human interest stories | English | 0/0 | https://www.usatoday.com/story/news/world/2020/01/25/coronavirus-grave-crisis-us-diplomats-ordered-leave-wuhan/4574189002/ |
| 80 | No | 0 | 63 | Commercial | Partly exclusive | Human interest stories | English | 0/0 | https://www.wired.com/story/would-the-coronavirus-quarantine-of-wuhan-even-work/ |
| 81 | No | 0 | 26 | News | Partly exclusive | Human interest stories | English | 0/0 | <https://english.manoramaonline.com/news/kerala/2020/01/26/lessons-kerala-can-learn-from-wuhan-as-it-battles-coronavirus.html> |
| 82 | No | 0 | 34 | News | Partly exclusive | Medical facts | English | 0/0 | <https://en.yna.co.kr/view/aen20200126000700320> |
| 83 | No | 0 | 38 | News | Exclusive | Medical facts, human interest stories | English | 0/0 | https://www.africanews.com/2020/02/05/ivory-coast-tests-suspected-coronavirus-case/ |
| 84 | No | 2 | 27 | News | Partly exclusive | Medical facts, human interest stories, question and answer | English | 0/0 | https://www.forbes.com/sites/judystone/2020/01/11/wuhan-coronavirus-outbreak-shows-the-importance-of-sound-science-sleuthing-and-cooperation/ |
| 85 | No | 1 | 35 | News | Exclusive | Human interest stories, question and answer, medical facts | English | 0/0 | https://www.theglobeandmail.com/world/article-the-wuhan-coronavirus-what-we-know-so-far-about-the-new-disease-from/ |
| 86 | No | 0 | 29 | Commercial | Partly exclusive | Medical facts, human interest stories | English | 0/0 | <https://www.spglobal.com/platts/en/market-insights/latest-news/metals/012420-wuhan-coronavirus-dampens-sentiment-in-chinas-steel-markets> |
| 87 | No | 3 | 45 | Commercial | Exclusive | Medical facts, human interest stories | English | 0/0 | <http://www.sixthtone.com/news/1005112/wuhan-coronavirus-latest-updates> |
| 88 | No | 0 | 25 | News | Partly exclusive | Medical facts, human interest stories | English | 0/0 | <https://www.rfa.org/english/news/china/wuhan-outbreak-01092020133656.html> |
| 89 | No | 1 | 29 | News | Exclusive | Medical facts, human interest stories | English | 0/0 | <https://apnews.com/14d7dcffa205d9022fa9ea593bb2a8c5> |
| 90 | No | 1 | 37 | Commercial | Partly exclusive | Medical facts, human interest stories | English | 0/0 | <https://news.yahoo.com/west-blames-wuhan-coronavirus-china-101401332.html> |
| 91 | No | 0 | 34 | Non-profit organization | Partly exclusive | Medical facts, human interest stories | English | 0/0 | https://khn.org/news/flu-far-deadlier-than-wuhan-virus/ |
| 92 | No | 0 | 22 | News | Exclusive | Medical facts, human interest stories | English | 0/0 | <https://www.thestandard.com.hk/breaking-news/section/3/140403/two-quarantined-in-zhejiang-for-wuhan-coronavirus> |
| 93 | No | 0 | 24 | News | Partly exclusive | Human interest stories | English | 0/0 | https://www.theolivepress.es/spain-news/2020/01/22/spain-preparing-for-wuhan-coronavirus-as-thousands-of-chinese-set-to-visit-for-their-new-year-holidays/ |
| 94 | No | 1 | 28 | University/medical center, non-profit organization | Partly exclusive | Medical facts, human interest stories | English | 0/0 | https://www.healthpolicy-watch.org/mystery-virus-in-wuhan-identified-as-novel-coronavirus-researchers-still-searching-for-animal-host/ |
| 95 | No | 0 | 62 | University/medical center | Exclusive | Medical facts, question and answer, human interest stories | English | 0/0 | https://www.idsociety.org/public-health/wuhan-coronavirus/ |
| 96 | No | 0 | 32 | Commercial | Partly exclusive | Human interest stories, medical facts | English | 0/0 | <https://news.sky.com/story/china-virus-britons-advised-against-travel-to-outbreak-epicentre-of-wuhan-11915188> |
| 97 | No | 0 | 25 | News | Exclusive | Human interest stories, medical facts | English | 0/0 | <https://www.scmp.com/news/china/society/article/3047278/wuhan-goes-shutdown-china-tries-contain-deadly-coronavirus> |
| 98 | No | 0 | 20 | News | Exclusive | Human interest stories, medical facts | English | 0/0 | <https://bigthink.com/politics-current-affairs/wuhan-coronavirus> |
| 99 | No | 2 | 26 | University/medical Center | Partly exclusive | Human interest stories | English | 0/0 | https://www.technologyreview.com/s/615124/coronavirus-china-wuhan-hong-kong-misinformation-censorship/ |
| 100 | No | 0 | 25 | Commercial | Partly exclusive | Human interest stories | English | 0/0 | <https://english.kyodonews.net/news/2020/02/7582dd522634-urgent-3-more-returnees-from-wuhan-test-positive-for-new-coronavirus-in-japan.html> |
| 101 | No | 0 | 32 | Commercial | Exclusive | Human interest stories, medical facts | English | 0/0 | <https://alertnation.blogspot.com/2020/01/coronavirus-wuhan-china.html> |
| 102 | No | 0 | 20 | News | Partly exclusive | Human interest stories | English | 0/0 | <http://www.surinenglish.com/local/202001/31/coronavirus-fears-increase-wuhan-20200131094131-v.html> |
| 103 | No | 0 | 24 | Commercial | Partly exclusive | Human interest stories | English | 0/0 | <https://www.channelnewsasia.com/news/commentary/china-wuhan-virus-lockdown-travel-flight-bus-quarantine-new-year-12372790> |
| 104 | No | 0 | 29 | News | Exclusive | Human interest stories | English | 0/0 | https://www.independent.co.uk/news/world/aasi/coronavirus-news-live-china-latest-death-toll-symptoms-wuhan-virus-uk-a9307271.html |
| 105 | No | 0 | 27 | News | Exclusive | Human interest stories, medical facts | English | 0/0 | https://www.alwihdainfo.com/wuhan-takes-efforts-to-combat-novel-coronavirus-at-community-level_a82235.html |
| 106 | No | 0 | 23 | News | Partly exclusive | Human interest stories | English | 0/0 | https://newsy-today.com/coronavirus-china-cuts-all-ties-to-wuhan-politics/ |
| 107 | No | 0 | 24 | News | Exclusive | Human interest stories | Spanish | 0/0 | https://www.aa.com.tr/es/mundo/se-confirma-el-primer-caso-de-coronavirus-en-b%c3%a9lgica/1724122 |
| 108 | No | 2 | 19 | Commercial | Exclusive | Medical facts, human interest stories | Spanish | 0/0 | http://www.juventudrebelde.cu/internacionalei/2020-02-04/oms-asegura-que-coronavirus-de-wuhan-aun-no-es-una-pandemia-y-no-ha-mutado |
| 109 | No | 1 | 23 | News | Partly exclusive | Medical facts | English | 0/0 | https://www.scotsman.com/news/people/whiwhi-cured-my-coronavirus-says-wuhan-based-brit-who-contracted-deadly-flu-1-5084964 |
| 110 | No | 2 | 26 | Commercial | Partly exclusive | Human interest stories | English | 0/0 | https://aldianews.com/articles/cultura/health/latinos-wuhan-epicenter-coronavirus/57500 |
