# Supplementary material for: Misinformation of COVID-19 on the Internet: Infodemiology Study
Source: JMIR Public Health Surveill. 2020 Apr 9;6(2):e18444. doi: 10.2196/18444 (PMC7147328; doi:10.2196/18444)
Supplement: Multimedia Appendix 2 [file publichealth_v6i2e18444_app2.docx]

Table 2. Analysis of the first 50 websites using the Google search engine, compared to medical literature to determine the information veracity.

| **No.** | **Main idea or direct citation** | **Comparison to medical literature** | **Reference** | **True/ Partially true/ False** | **Link** |
| --- | --- | --- | --- | --- | --- |
| 1 | Much is still unknown about the virus, including two factors that are pivotal to predicting the global human toll: how easily the virus spreads from person to person, and how many of those infected develop severe disease that can lead to death. | Every infected patient spreads the virus to 2.2 people. Around 11% of infected people died. | Riou J, 2020., Chen N, 2020 ^17,18^ | Partially true | https://www.nytimes.com/article/what-is-coronavirus.html |
| 2 | How can people protect themselves? Wash hands thoroughly with soap  Cover your face when coughing and sneezing. Face masks optional-scientists doubt their effectiveness. | The most effective control measures to reduce transmission, has not been identified yet. | Li Q, 2020 ^19^ | Partially true | <https://www.aljazeera.com/news/2020/01/china-coronavirus-death-toll-surges-latest-updates-200131232932230.html> |
| 4 | Its genetic code is formed by one single RNA chain, classifying it as a positive single chain RNA virus. | Coronavirus possess a single strand, positive-sense RNA genome ranging from 26 to 32 kilobases. | Lu R, 2020 ^20^ | True | https://es.wikipedia.org/wiki/Coronavirus_de_Wuhan |
| 5 | The [incubation period](https://en.wikipedia.org/wiki/Incubation_period) (time from exposure to onset of symptoms) ranges from 2 to 14 days. | The mean incubation period is estimated to be 5.2 days | Li Q, 2020 ^19^ | Partially true | <https://en.wikipedia.org/wiki/2019%E2%80%9320_Wuhan_coronavirus_outbreak> |
| 6 | The government has launched a public health campaign urging people to use tissues when they cough or sneeze and to wash their hands regularly. | 2 | Li Q, 2020 ^19^ | Partially true | <https://www.bbc.com/news/health-51345279> |
| 7 | Antibiotics do not work against viruses, they only work on bacterial infections. The novel coronavirus is a virus and, therefore, antibiotics should not be used as a means of prevention or treatment. | Antibiotics don't work on viral infections, but some patients infected with coronavirus may require them when secondary bacterial infection occurred. | Chen N, 2020 ^18^ | Partially true | <https://www.who.int/emergencies/diseases/novel-coronavirus-2019> |
| 8 | The virus, which may have [jumped from animals to people at a market](https://www.businessinsider.com/wuhan-coronavirus-sars-bats-animals-to-humans-2020-1) in the city of Wuhan, has killed 259 people and infected at least 12,000. | On December 29, 2019, the first 4 cases reported, all linked to the Huanan (Southern China) Seafood Wholesale Market, were identified by local hospitals using a surveillance mechanism for “pneumonia of unknown etiology” that was established in the wake of the 2003 severe acute respiratory syndrome (SARS) outbreak with the aim of allowing timely identification of novel pathogens such as 2019- nCoV. | Li Q, 2020 ^19^ | True | <https://www.businessinsider.com/wuhan-coronavirus-research-studies-published-2020-1> |
| 9 | Restricting person-to-person contact is the most effective way of preventing this spreading. | 2 | Li Q, 2020 ^19^ | Partially true | <https://www.theguardian.com/world/live/2020/jan/29/coronavirus-live-updates-china-wuhan-death-toll-cases-symptoms-treatment-evacuation-us-japanese-citizens-latest-news> |
| 11 | There are no approved treatments for coronaviruses, but on Feb. 6, China started enrolling a small number of patients in a [clinical trial of remdesivir](https://clinicaltrials.gov/ct2/show/NCT04257656), an experimental antiviral made by American pharmaceutical company Gilead that has not yet been approved for any use, but has shown promise in lab studies. | There is no clinical practice guide for this infection, nevertheless, some authors report good outcomes using some of the next: Oxygen therapy, mechanical ventilation, continuous renal replacement therapy, extracorporeal membrane oxygenation, antibiotic therapy, antifungal therapy, antiviral treatment (oseltamivir, ganciclovir, lopinavir, ritonavir), glucocorticoids and intravenous immunoglobulin therapy | Chen N, 2020 ^18^ | False | https://www.cnet.com/how-to/coronavirus-cases-pass-11000-us-declares-emergency-everything-we-know/ |
| 12 | A coronavirus is a virus that is found in animals. Sometimes these viruses can be transmitted from animals to humans, although it is rare | Coronaviruses can cause multiple system infections in various animals and mainly respiratory tract infections in humans, such as SARS and MERS. Most patients have mild symptoms and good prognosis | Chen N, 2020 ^18^ | True | https://familydoctor.org/condition/coronavirus/ |
| 14 | Less commonly reported symptoms include sputum production, headache, hemoptysis, and diarrhea. | Some authors have reported headache, sputum production and diarrhea as less common symptoms. Hemoptysis have not been reported. | Chen N, 2020., Wang D, 2020^18, 21^ | Partially true | https://www.cdc.gov/coronavirus/2019-ncov/index.html |
| 15 | Young children, elderly people and those with underlying conditions ( hypertension, heart disorders, diabetes, liver disorders, and respiratory disease) are expected to be more at risk of developing severe symptoms. | Old age, obesity, and presence of comorbidity might be associated with increased mortality | Chen N, 2020 ^18^ | True | https://www.ecdc.europa.eu/en/novel-coronavirus-china |
| 16 | According to the [WHO](https://www.who.int/emergencies/diseases/novel-coronavirus-2019/situation-reports), 16-21% of people with the virus in China became severely ill and 2-3% of those infected have died. | Some authors reported 23-26% of infected patients had to enter to Intensive care unit. Others reported around 11% of the infected patients died. | Chen N, 2020., Wang D, 2020 ^18, 21^ | False | <http://theconversation.com/how-contagious-is-the-wuhan-coronavirus-and-can-you-spread-it-before-symptoms-start-130686> |
| 17 | Most coronaviruses result in mild symptoms, including upper-respiratory tract infections like the common cold, [according to](https://www.cdc.gov/coronavirus/about/symptoms.html) the U.S. Centers for Disease Control and Protection (CDC). | A study showed only 31% of patients infected were discharged from hospital, the rest remained at the hospital or at intensive care unit at the end of the study. | Chen N, 2020 ^18^ | False | https://time.com/5759289/wuhan-pneumonia-outbreak-disease/ |
| 19 | Health officials have determined that this novel strain of the coronavirus respiratory illness capable of spreading through human-to-human contact, droplets carried through sneezing and coughing, and germs left on inanimate objects | This virus can spread to human-to-human contact. Nevertheless, medical bibliography does not specify what kind of contact may infect other people. | Li Q, 2020., Chan JF, 2020 ^19, 22^ | Partially true | https://geoawesomeness.com/track-china-coronavirus-real-time-map-global-cases-death-wuhan/ |
| 20 | It is far too simplistic to take the 130 deaths and the 6,000 cases and come up with a death rate of 2% | Some authors reported around 11% of the infected patients died. | Chen N, 2020 ^18^ | True | https://www.bbc.co.uk/news/uk-51292590 |
| 21 | Coronavirus are microorganisms that can easily mutate. | This virus mutates on every replication | Lu R, 2020 ^20^ | True | https://elpais.com/elpais/2020/01/28/inenglish/1580220348_402354.html |
| 23 | The seafood market in Wuhan, central China's Hubei Province, may not be the only source of the novel coronavirus (2019-nCoV) outbreak, according to a Chinese pulmonary disease specialist. | The Wuhan Seafood Wholesale Market has been proposed as the source of the novel coronavirus | Chen N, 2020., Li Q, 2020., Lu R, 2020., Wang D, 2020 ^18-21^ | Partially true | http://www.xinhuanet.com/english/2020-01/29/c_138741063.htm |
| 24 | It is also adapting and mutating, which could make the virus spread faster and possibly more difficult to treat. | This virus mutates on every replication. Nevertheless, no author has related these mutations to difficult on treatment. | Lu R, 2020 ^20^ | Partially true | https://www.dw.com/en/coronavirus-everything-you-need-to-know/a-52102486 |
| 25 | The disease is transmitted from human to human. | 19 | Li Q, 2020., Chan JF, 2020 ^19,22^ | True | https://www.rivm.nl/en/novel-coronavirus-in-china |
| 27 | As British researchers said a vaccine breakthrough could be just weeks away | There is no information about the development of a vaccine. |  | False | https://www.telegraph.co.uk/news/2020/01/25/british-citizens-wuhan-left-dark-foreign-office-coronavirus/ |
| 28 | The symptoms of the 2019 novel coronavirus, which can include fever and cough, are similar to other respiratory infections, including influenza. | It is true that symptoms include fever, cough and respiratory distress | Hui DS, 2020 ^23^ | True | https://www.ontario.ca/page/2019-novel-coronavirus-2019-ncov |
| 29 | Coronaviruses are a family of viruses that cause infections in the respiratory system. There are seven known coronaviruses, including the newly identified 2019-nCoV. | 12 | Zhu N, 2020 ^24^ | True | https://www.forbes.com/sites/leahrosenbaum/2020/01/23/everything-you-need-to-know-about-the-wuhan-coronavirus-outbreak/ |
| 30 | First identified in Wuhan in mid-December, the novel coronavirus (2019-nCoV) is in the same family of infections as severe acute respiratory syndrome (SARS) and Middle East respiratory syndrome (MERS) | 12 | Zhu N, 2020 ^24^ | True | https://www.cnn.com/2020/01/22/asia/china-wuhan-coronavirus-deadly-intl-hnk/index.html |
| 31 | For other coronaviruses, it can take anywhere from 2 to 14 days for a person exposed to the virus to have symptoms. | We estimated the mean incubation period to be 5.2 days | Li Q, 2020 ^19^ | True | https://multco.us/health-officer/wuhan-coronavirus |
| 35 | People can reduce the risk of acquiring and spreading respiratory infections by practicing good respiratory hygiene, such as: avoiding direct hand contact with your eyes, nose and mouth, maintaining good hand hygiene; washing hands with soap and water or alcohol hand sanitizer, after coughing or sneezing, after going to the toilet, and prior to eating and drinking and when coughing or sneezing cover your nose and mouth with disposable tissues and disposing of them in nearest waste bin after use. | 2 | Li Q, 2020 ^19^ | Partially true | https://www.ed.ac.uk/health-safety/guidance/communicable-infectious-diseases/wuhan-coronavirus |
| 36 | The virus is thought to have crossed to humans from bats | Virus from the family of coronavirus can be transmitted directly to humans from civets and dromedary camels, and viruses may originate in bats, but the origin of 2019-nCoV needs further investigation. | Chen N, 2020 ^18^ | Partially true | https://www.weforum.org/agenda/2020/01/wuhan-coronavirus-china-cepi-vaccine-davos/ |
| 37 | Doctors and scientists are still researching and debating the possible origin of the previously unknown Wuhan coronavirus, a respiratory virus that infects the lungs and can lead to pneumonia. | 36 | Chen N, 2020 ^18^ | True | https://globalvoices.org/specialcoverage/how-will-the-wuhan-coronavirus-impact-the-political-future-of-china/ |
| 38 | Coronavirus presents with flu-like symptoms including a fever, a cough, or difficulty breathing. The current evidence is that most cases appear to be mild. | It is true that symptoms include fever, cough and respiratory distress | Hui DS, 2020 ^23^ | True | https://phw.nhs.wales/news/public-health-wales-response-to-outbreak-in-wuhan-china/ |
| 40 | Scientists don’t even need to grow the virus in cells anymore. They can directly detect extremely small amounts of viral DNA in a patient’s spit or blood. | Detection of nucleic acids from fluids such as bronchoalveolar-lavage fluid is possible | Zhu N, 2020 ^24^ | True | https://www.voanews.com/science-health/new-tech-sharp-docs-made-fast-id-wuhan-coronavirus-possible |
| 41 | At the moment, there is no treatment at all, because this is a virus, antibiotics don’t work, they work only against bacteria, not viruses | 11 | Chen N, 2020 ^18^ | False | https://www.asiatimes.com/2020/01/opinion/how-china-failed-to-manage-wuhan-coronavirus/ |
| 44 | The 2019-nCov is transmitted from person to person and through the airways, when coughing or sneezing. | 19 | Li Q, 2020., Chan JF, 2020 ^19, 20^ | Partially true | https://www.practicaespanol.com/en/13-clear-things-about-wuhans-coronavirus-in-china/ |
| 45 | On average, each case infected 2.6 (uncertainty range: 1.5-3.5) other people up to 18th January 2020, based on an analysis combining our past estimates of the size of the outbreak in Wuhan with computational modelling of potential epidemic trajectories | R0 of coronavirus is estimated to be around 2.2, meaning that every patient infects 2.2 people. | Riou J, 2020 ^17^ | True | https://www.imperial.ac.uk/mrc-global-infectious-disease-analysis/news--wuhan-coronavirus/ |
| 47 | Hand Sanitizer should be placed at all offices, classrooms, public spaces, dormitories, restaurants, and restrooms, etc. Special attention of sanitizing should be given to locations and places such as elevators, stairway handrails, desk top, and computer keyboard, etc. | 2 | Li Q, 2020 ^19^ | Partially true | https://www.ntu.edu.tw/english/spotlight/2020/1797_20200130.html |
| 49 | Hong Kong has also instructed government employees to work from home for the rest of the week and requested private companies do the same | 2 | Li Q, 2020 ^19^ | Partially true | http://ba.n1info.com/English/NEWS/a406863/Wuhan-coronavirus-continues-its-global-spread-with-more-than-6-000-cases.html |
| 50 | Officials are unsure how likely people who get it are to die, whether it can be transmitted before people show symptoms, where it originated and when exactly it began circulating. | Old age, obesity, and presence of comorbidity might be associated with increased mortality. There is no clear about information about whether the virus can be transmitted before the onset of symptoms or not. The Huanan Seafood Wholesale Market has been proposed as the main source of the novel coronavirus. The first coronavirus outbreak began on December of 2019. | Chen N, 2020., Li Q, 2020., Lu R, 2020., Wang D, 2020 ^18-21^ | Partially true | https://www.latimes.com/science/story/2020-01-28/wuhan-chinas-coronavirus-50-million-people-quarantined |
